# Supplementary material for: The first Australian plant foods at Madjedbebe, 65,000–53,000 years ago
Source: Nat Commun. 2020 Feb 17;11:924. doi: 10.1038/s41467-020-14723-0 (PMC7026095; doi:10.1038/s41467-020-14723-0)
Supplement: Supplementary file 1 — Supplementary Information [file 41467_2020_14723_MOESM1_ESM.pdf]

Supplementary Information

The first Australian plant foods at Madjedbebe, 65,000–53,000 years ago

Florin et al.

Table of Contents

Supplementary Note 1: Identification of plant macrofossils
Supplementary Table 1: Madjedbebe Phase 2 plant macrofossil assemblage
Supplementary Figure 1: Age-depth curve model, comparing optically stimulated luminescence (OSL) and AMS radiocarbon dates
Supplementary Figure 2: Madjedbebe rock shelter
Supplementary Figure 3: *Buchanania* sp. identification proof
Supplementary Figure 4: *Canarium australianum* identification proof
Supplementary Figure 5: *Pandanus* sp. identification proof
Supplementary Figure 6: *Persoonia falcata* identification proof
Supplementary Figure 7: *Terminalia* sp. identification proof
Supplementary Figure 8: Arecaceae family sheath/pith identification proof
Supplementary References

Supplementary Note 1: Identification of plant macrofossils

Buchanania sp. endocarp

Buchanania genus endocarp are biconvex in shape, their internal seed locule created by the conjoining of two convex discs at their circumference. The outer surface of their endocarp is mildly striated and patterned by small isodiametric cells (Supplementary Fig. 3a-d). The internal anatomy of the endocarp is dense and its transverse section is made up of vertically-banded sclerenchyma (Supplementary Fig. 3e-f).

There are two species from the *Buchanania* genus in the Northern Territory: *B. obovata* and *B. arborescens*[1]. Their fruit are similar in size (between 8mm and 15mm in diameter), although that of *B. arborescens* is slightly smaller. The fragmentary endocarp remains in Phase 2 at Madjedbebe are not distinguishable to species level.

Canarium australianum endocarp

Canarium spp. endocarp are obovoid to ellipsoid in shape (Supplementary Fig. 4a, b). They have three conjoining seed chambers, with one developed to maturity. The inner surface of their endocarp has a thin layer of tissue, which when removed is underlain by small hexagonal cells (Supplementary Fig. 4c-d). The internal anatomy, in transverse section, has two layers: the outer consisting of uniform thick-walled isodiametric sclerenchyma cells; and the inner consisting of vertically elongated sclerenchyma cells, forming a slightly waved pattern.

There is only one species of *Canarium* in the Northern Territory today, *C. australianum*[1]. Whilst New Guinea, once northern Sahul, is home to several other species, the Pleistocene would likely not have been wet enough to aid in the extension of the distribution of these woodland- and rainforest-based species across the savannah-dominated Arafura Shelf[2-5]. The size of the archaeological fragments of endocarp are also in keeping with *C. australianum*.

Polydrupe *Pandanus* spp. and *Pandanus spiralis* drupes

Polydrupe *Pandanus* spp. drupes are large ovoid structures (Supplementary Fig. 5e), their multiple seed locules (endocarp, Supplementary Fig. 5a, b) grouped centrally within less dense fibrous tissue (mesocarp, Supplementary Fig.5c, d). Archaeologically it is the denser endocarp that most commonly preserves. This tissue consists of closed collateral vascular bundles, surrounded in the transverse section by flaring ground tissue (Supplementary Fig. 5b). Whereas monodrupe *Pandanus* spp. have thinner locule walls and more evenly spaced vascular bundles, the vascular bundles of the polydrupe *Pandanus* spp. are randomly spaced. All the archaeological specimens from Phase 2 are in keeping with the latter anatomy.

There are two species of polydrupe *Pandanus* in the Northern Territory, *P. spiralis* and *P. basedowii*[1]. In most cases the fragmented nature of the archaeological specimens prevents them from being speciated. However, the mesocarp of the *P. spiralis* has a distinct 'starburst' appearance, the fibrous bundles enclosed concentrically by elongated parenchyma cells (Supplementary Fig. 5c, d). This 'starburst' anatomy is visible in one of the fragments from the Phase 2 assemblage (Supplementary Fig. 5f).

<u>*Persoonia falcata* endocarp</u>
Persoonia spp. endocarp are obovoid in shape, enclosing a single seed locule (Supplementary Fig. 6a, b). Their inner surface is patterned by elongated, rectangular cells (Supplementary Fig. 6c, d). The transverse section consists solely of uniform thick-walled isodiametric sclerenchyma.

Persoonia falcata is the only representative of this genus in the Northern Territory[1].

<u>*Terminalia* spp. endocarp</u>
Terminalia spp. endocarp are laterally-compressed, ellipsoid and apiculate in shape, their internal seed locule created by the conjoining of two elliptical sides. Their outer surface is striated like an olive (Supplementary Fig. 7b). However, when weathered it loses this patterning and exposes a series of holes running perpendicularly through the upper portion of the endocarp (Supplementary Fig. 7a, b). The transverse section is made up of flaring sclerenchyma (Supplementary Fig. 7c, d).

There are at least 17 species of *Terminalia* in the Northern Territory[1]. Their fruits ranging in size from approximately 10mm (*T. microcarpa*) to 65mm in length (*T. catappa*). The fragmentary endocarp remains in Phase 2 at Madjedbebe are not distinguishable to species level. However, they likely come from species with medium-sized fruits and endocarps (e.g. *T. ferdinandiana*, *T. grandiflora*).

<u>Arecaceae cf. *Livistona* spp. stem</u>
Arecaceae cf. *Livistona* spp. stem tissue is characterised by its range of reniforma fibrovascular bundles (Supplementary Fig. 8a-f). While most of these fibrovascular bundles have two or more metaxylem elements, some only have one. The occurrence of fibrovascular bundles with only one metaxylem element is more frequent within the central zone of the stem. In the charred archaeological fragments, the metaxylem and protoxylem are well-preserved, whereas the phloem is in most part only identifiable by a fracture plane (Supplementary Fig. 8c, e). Globular echinate and globular granulate phytoliths occur along the fibrovascular bundles and fibrous bundles in both the central zone and the subcortical zone.

There are six species of *Livistona*, *L. benthamii, L. humilis, L. inermis*, *L. lorophylla*, *L. mariae* and *L. victoriae*, and a further eight genera (nine species) from the Areceaeae family in the Northern Territory[1]. The anatomy of Arecaceae stem Type A is consistent with *Livistona* spp. within the modern reference collection. However, there is a considerable degree of overlap between closely related genera of Arecaceae[6], and *Livistona* and two other genera (*Corypha* and *Cocos*) found in the Northern Territory belong to the Coryphoideae subfamily of Arecaceae. Therefore, this identification can only be considered tentative without further reference material, especially when considering the fragmentary nature of the plant macrofossils from Phase 2.

Supplementary Table 1: Madjedbebe Phase 2 plant macrofossil assemblage.

Plant part	Taxa/type	C2/46	C2/45	C2/44	C2/43	C2/42	C2/41	C2/40	C2/39A	C2/38A	C2/38	C2/37A	C2/37	C1/43A
**Endocarp**	*Buchanania* sp.	1	0	0	0	0	0	0	0	0	0	0	1	0
	cf. *Buchanania* sp.	0	0	0	0	0	1	0	0	0	0	2	0	0
	Canarium australianum	0	0	0	0	0	0	0	0	1	3	0	2	0
	cf. *Canarium australianum*	1	1	1	0	0	1	2	0	1	3	5	10	0
	Pandanus sp.	1	2	0	0	5	4	4	19	0	8	26	24	0
	cf. Pandanus sp.	0	0	0	0	0	0	1	3	4	3	0	4	0
	Persoonia falcata	0	0	1	0	1	0	0	0	0	0	1	1	0
	cf. *Persoonia falcata*	0	0	0	0	0	0	0	0	1	0	1	18	0
	Terminalia sp.	0	0	0	0	0	0	0	0	0	1	0	1	0
	Unidentified	5	0	6	1	10	7	23	54	41	61	67	142	0
**Mesocarp**	*Pandanus spiralis*	0	0	0	0	0	0	0	0	0	0	0	1	0
**USO tissue**	Secondary root tissue: Type A	0	0	0	0	0	0	0	1	0	0	0	0	0
	Monocot. stem tissue: Type A	0	2	0	0	0	1	0	0	0	0	0	0	0
	Monocot. stem tissue: Type B	0	0	0	0	0	0	3	1	1	0	1	5	0
**Palm stem tissue**	Arecaceae stem: Type A (cf. *Livistona* spp.)	0	0	2	0	0	0	0	0	1	0	0	0	0
	cf. Arecaceae stem: Type A (cf. *Livistona* spp.)	0	0	0	0	1	0	0	8	1	1	0	2	1
	Arecaceae stem: Type B	0	1	1	0	0	0	0	0	0	0	0	0	0
	cf. Arecaceae stem: Type B	2	1	0	0	0	0	0	0	0	0	0	0	0

**USO tissue/Palm stem tissue**	Unidentified monocot. stem tissue	2	0	0	1	0	0	0	1	0	0	0	5	0
	>20um parenchyma	1	0	1	0	0	3	0	39	19	12	11	63	7
**Other**	cf. Malvaceae seed	0	0	0	0	1	0	0	0	0	0	0	0	0
	<20um parenchyma	3	0	3	0	2	1	0	8	0	9	9	41	0
	Vitrified	1	0	1	0	3	2	8	24	22	31	35	52	2
	**Total NISP**	17	7	16	2	23	20	41	158	92	132	158	372	10

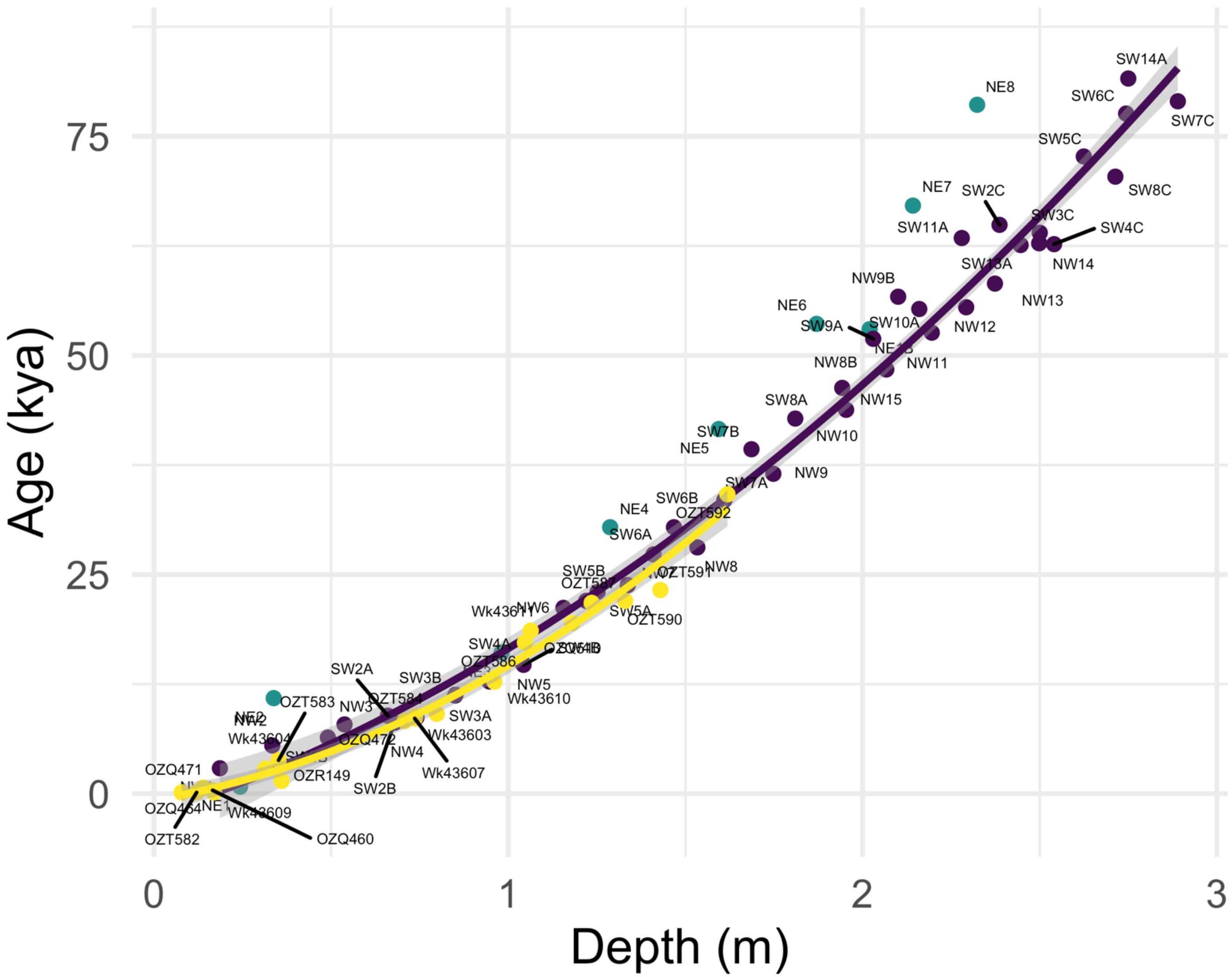

Age (kya)
Depth (m)
0
25
50
75
0
1
2
3
SW14A
NE8
SW6C
SW7C
SW5C
SW8C
NE7
SW2C
SW3C
SW4C
SW11A
NW14
NW9B
NW13
NE6
SW9A
SW10A
NW12
NW8B
NW11
SW8A
NW15
NW10
SW7B
NE5
NW9
NE4
SW6B
OZT592
SW6A
NW8
SW5B
OZT590
SW5A
SW4A
OZT586
NW5
Wk43610
SW2A
SW3B
OZT583
OZT584
SW3A
Wk43603
OZQ472
NW4
Wk43607
OZR149
SW2B
OZQ471
Wk43609
OZT582
OZQ460

Supplementary Figure 1: Age-depth curve model, comparing optically stimulated luminescence (OSL) and AMS radiocarbon dates. Age depth curve model, comparing OSL ages (in purple) from the northwest and southwest sides of Squares A, B and C to AMS radiocarbon ages (in yellow) from Squares B, C, D and E, 3-5 (excluding WK43606 and OZT593, see Supplementary Fig. 2c). Green points show OSL ages from the NE side for reference, these are not included in the statistical models. The age-depth models for C14 ages and OSL ages are not significantly different (ANOVA on linear models with quadratic terms, $F = 1.518$, $df = 3$, $p = 0.2195$). Data and R code for this figure are available online at http://doi.org/10.17605/OSF.IO/YDUZP[7].

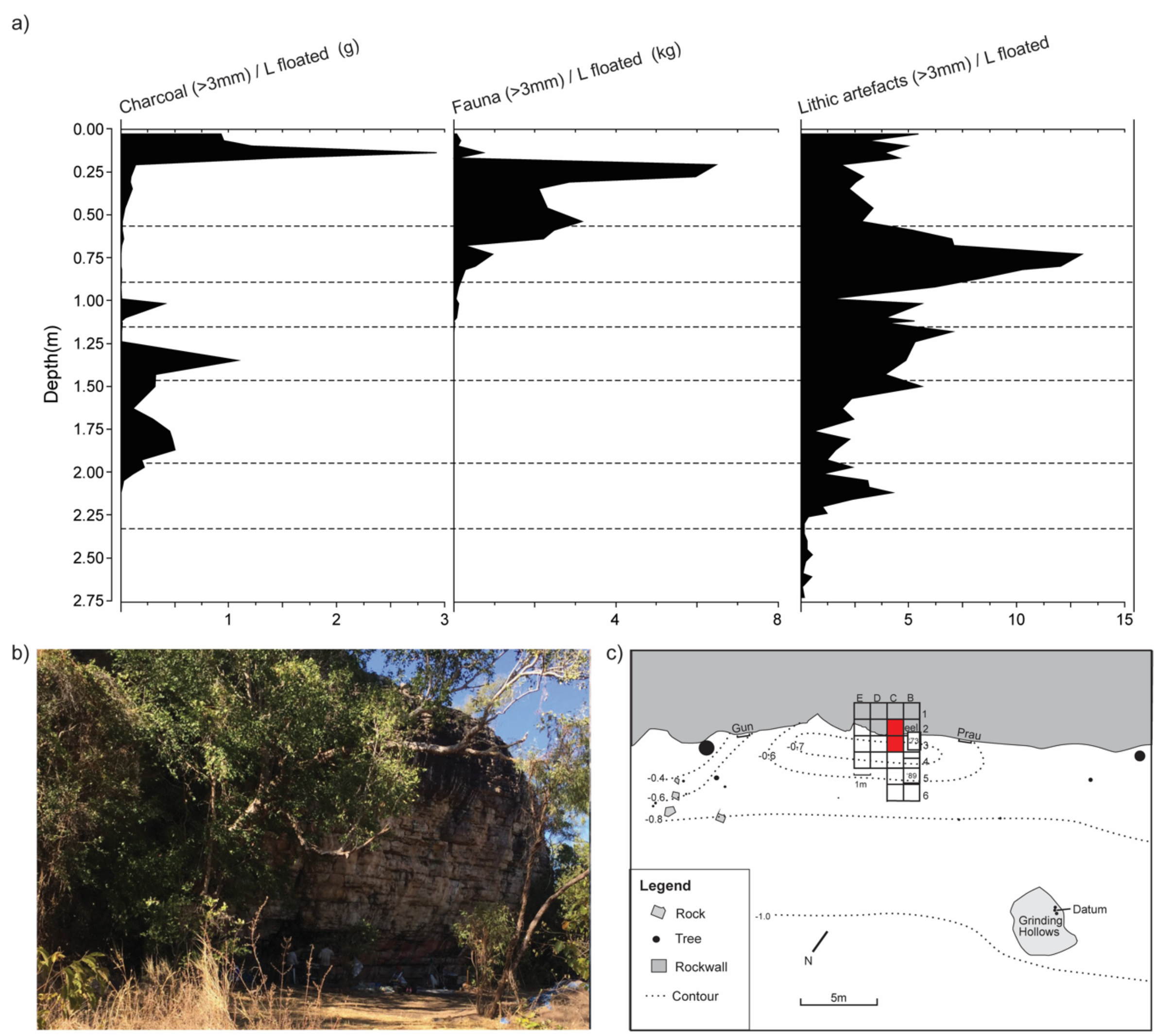

Supplementary Figure 2: Madjedbebe rock shelter. a) >3mm charcoal, fauna and lithic artefact discard rates from Square C2/C3, dashed lines demarcate the phase boundaries; b) photograph of the site during the 2015 excavation, photo taken by CC; c) site plan showing the 1972, 1989, 2012 and 2015 excavation squares, with Squares C2 and C3 coloured red, adapted from Clarkson et al. 2017[8].

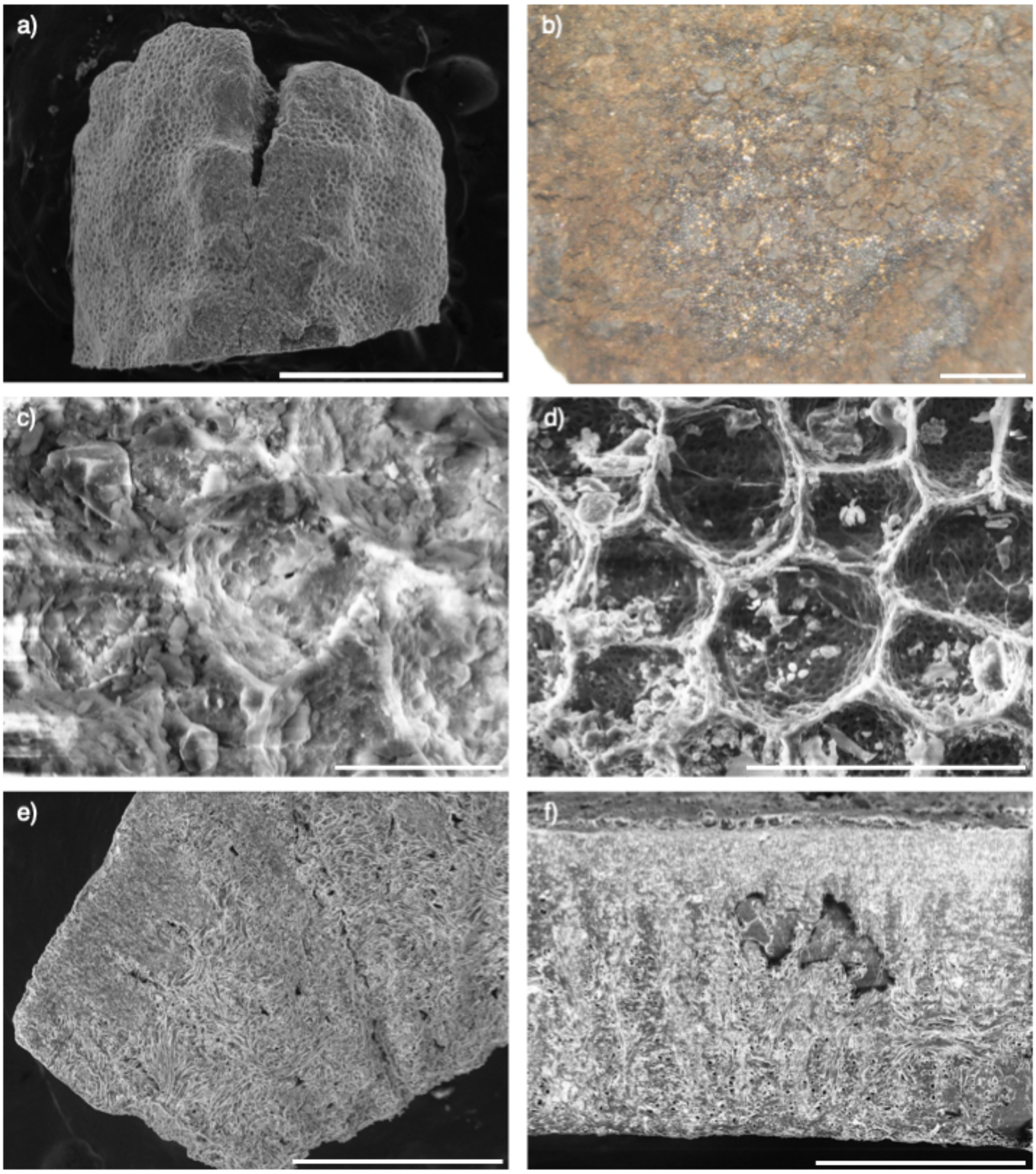

Supplementary Figure 3: *Buchanania* sp. identification proof, comparing an archaeological *Buchananaia* sp. endocarp fragment from C2/46(HR) (left) with a modern reference specimen of *Buchanania obovata* (UQM 3087) (right). a) the outer surface of the archaeological specimen, at the conjoin of the endocarp discs, displaying small isodiametric cell patterning, scale bar is 1mm; b) the outer surface of the modern reference specimen, displaying small isodiametric cell patterning, scale bar is 1mm; c) close-up of the isodiametric cells on the outer of the archaeological specimen, scale bar is 30μm; d) close-up of the isodiametric cells on the outer of the modern reference specimen, scale bar is 100μm; e) transverse section of the archaeological specimen, with vertically-banded sclerenchyma, scale bar is 500μm; f) transverse section of the modern reference specimen, with vertically-banded sclerenchyma, scale bar is 500μm.

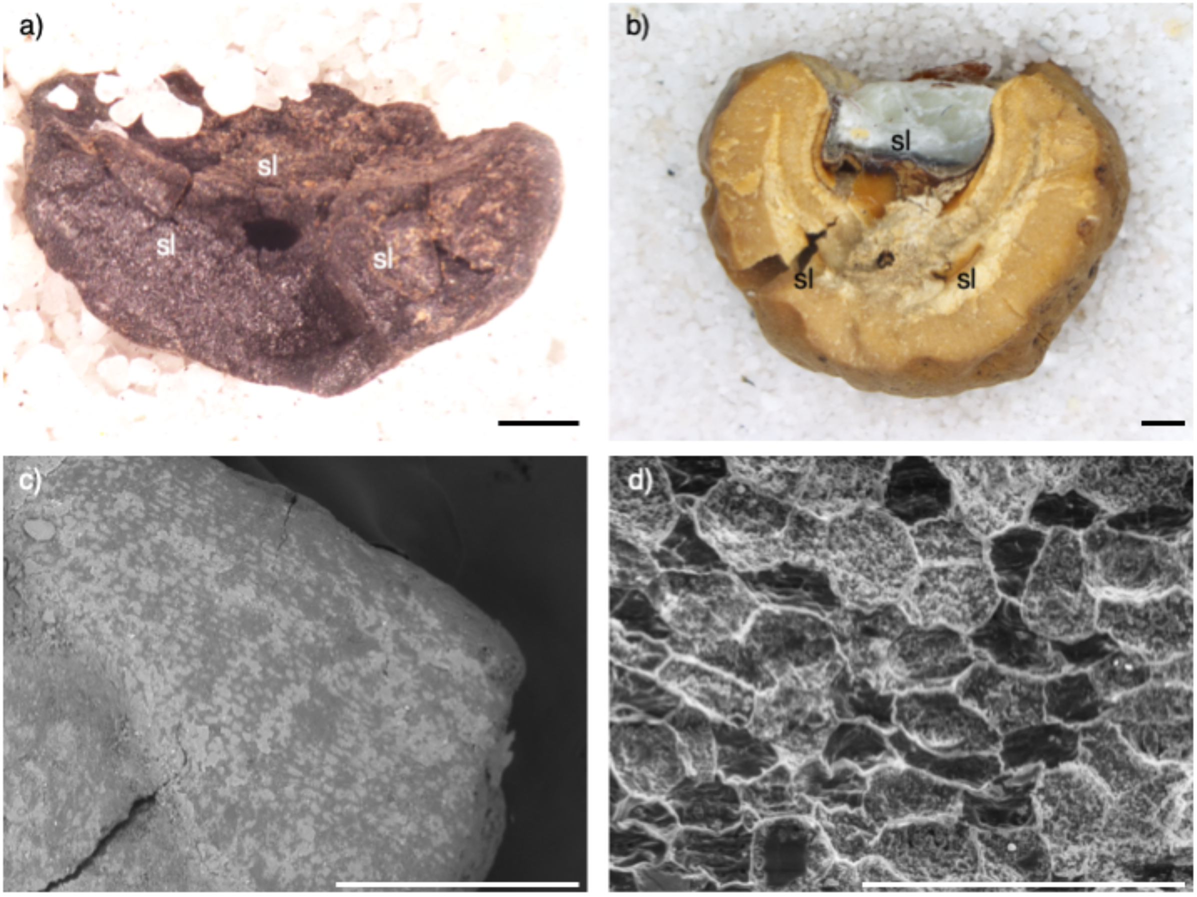

Supplementary Figure 4: *Canarium australianum* identification proof, comparing archaeological endocarp fragments from C2/38(HR) and C2/37(HR) (left) with a modern reference specimen (UQM 3104) (right; sl: seed locule). a) the endocarp of the archaeological specimen from C2/389(HR), with visible mature and immature seed locules, scale bar is 500μm; b) the endocarp of the modern reference specimen, with visible mature and immature seed locules; c) the worn internal surface of the archaeological specimen from C2/37(HR), with a still visible pattern of small hexagonal cells, scale bar is 500μm; d) The internal surface of the modern reference specimen, with small hexagonal cells, scale bar is 200μm.

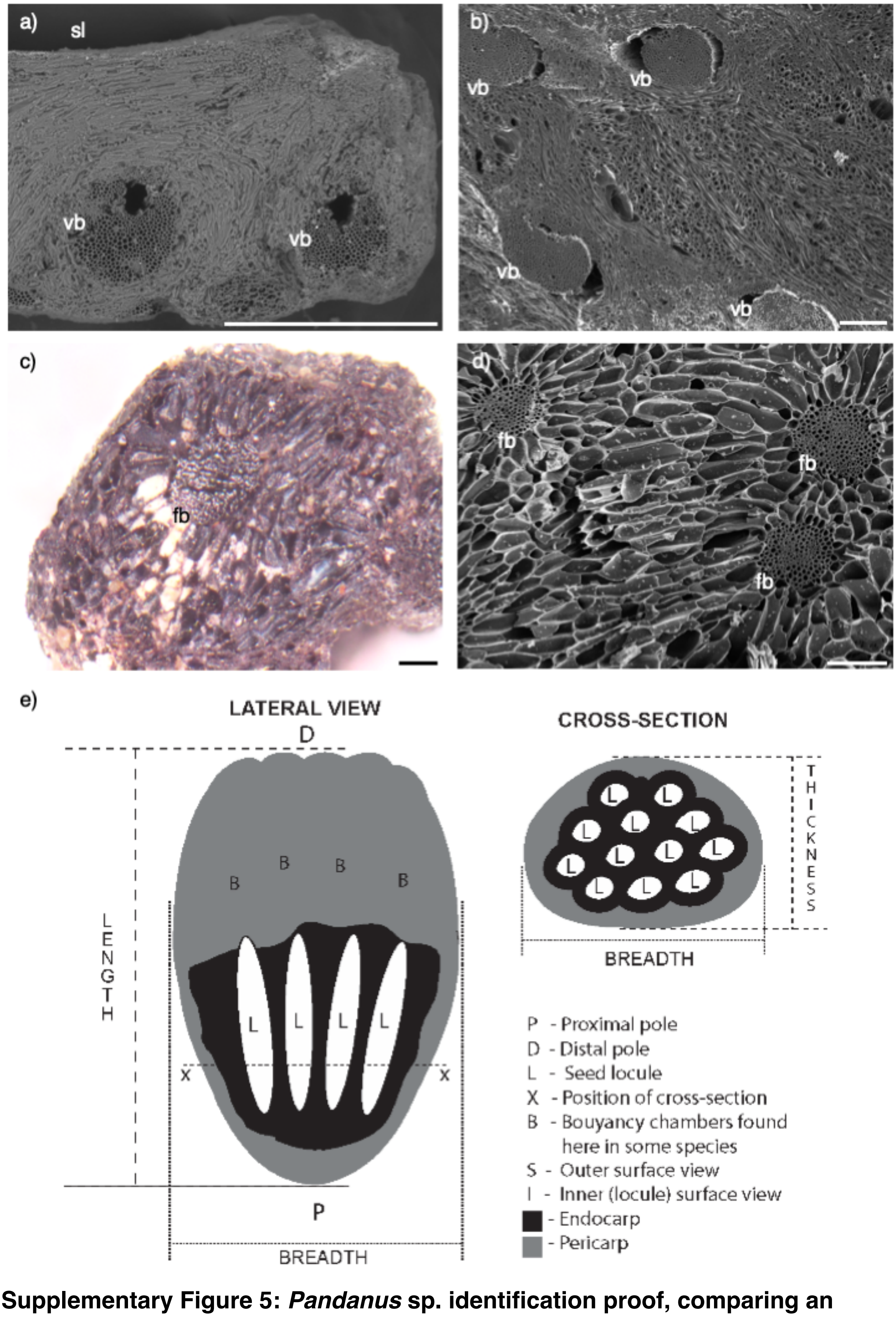

Supplementary Figure 5: *Pandanus* sp. identification proof, comparing an archaeological *Pandanus* sp. endocarp fragment from C2/41(HR) and an archaeological *Pandanus spiralis* pericarp fragment from C2/37 (left) with a

modern reference specimen (UQM2643) (right and bottom; sl: seed locule, vb: vascular bundle, fb: fibrous bundle). a) transverse section of the endocarp of the archaeological specimen from C2/41(HR), with flaring ground tissue, two closed collateral vascular bundles and a visible seed locule, scale bar is 500μm; b) transverse section endocarp of the modern reference specimen, with flaring ground tissue and several amphicribal vascular bundles, scale bar is 200μm; c) transverse section of the pericarp of the archaeological specimen from C2/37, displaying the characteristic 'starburst' anatomy of *Pandanus spiralis* pericarp, scale bar is 100μm; d) transverse section of the pericarp of the modern reference specimen), displaying the characteristic 'starburst' anatomy of *Pandanus spiralis* pericarp; e) schematic of a polydrupe pandanus drupe.

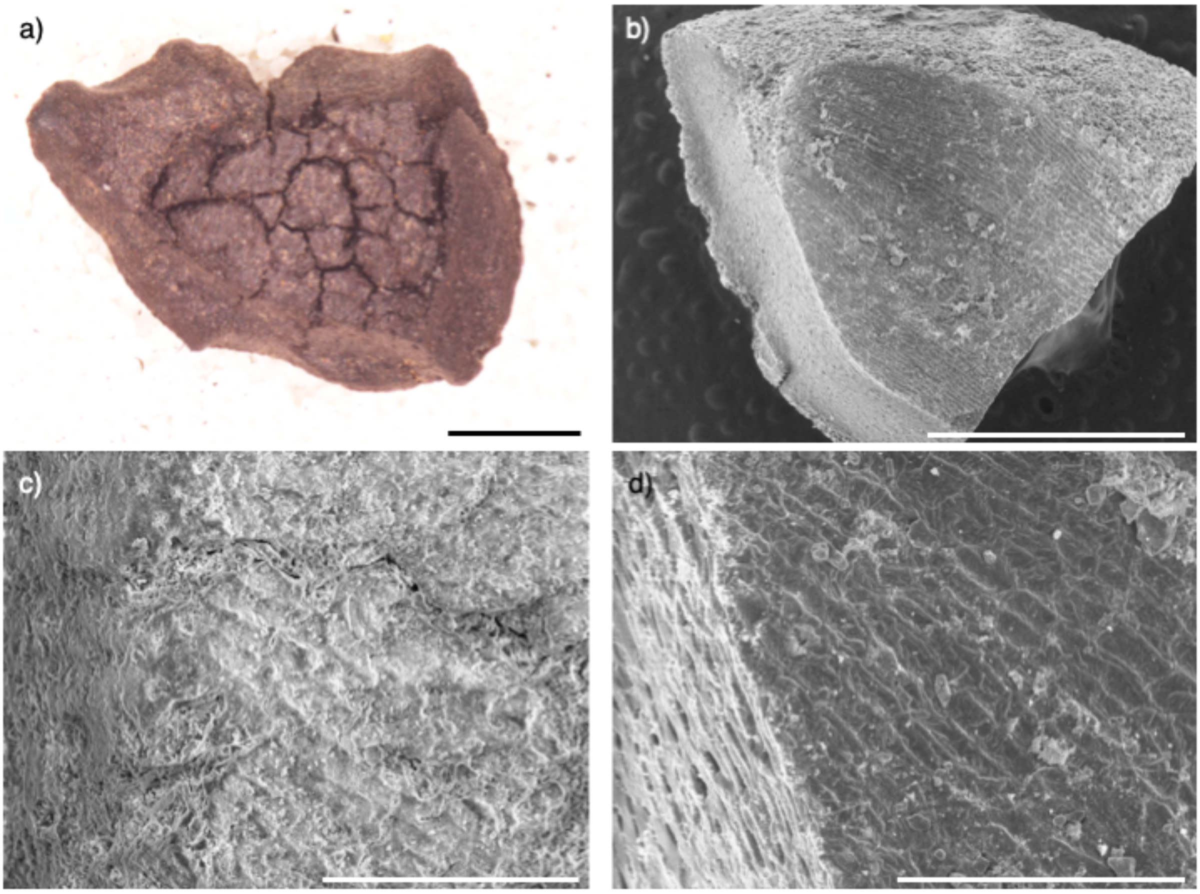

Supplementary Figure 6: *Persoonia falcata* identification proof, comparing an archaeological endocarp fragment from C2/37(HR) (left) with a modern reference specimen (UQM3087) (right). a) the endocarp of the archaeological specimen, scale bar is 1mm; b) the endocarp of the modern reference specimen, scale bar is 2mm; c) the worn internal surface of the archaeological specimen, with still visible elongated, rectangular cells, scale bar is 200μm; d) the internal surface of the modern reference specimen, with elongated, rectangular cells, scale bar is 300μm**.**

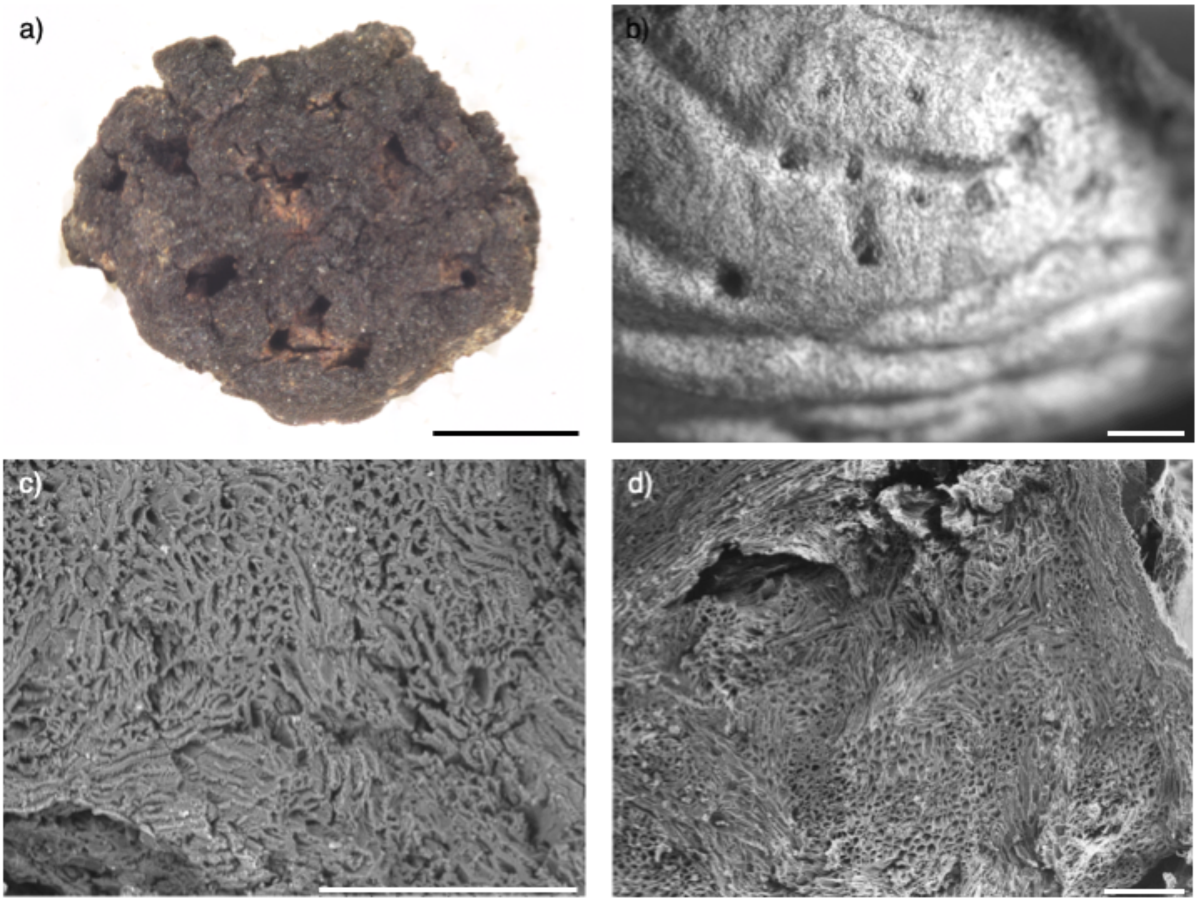

Supplementary Figure 7: *Terminalia* sp. identification proof, comparing an archaeological endocarp fragment from C2/37HR (left) with a modern reference specimen (UQM (right). a) the endocarp of the archaeological specimen, with surface deterioration and holes, scale bar is 1mm; b) the endocarp of the modern reference specimen, with holes where the surface has worn away, scale bar is 1mm; c) transverse section of the archaeological specimen, with flaring sclerenchyma, scale bar is 200μm; d) transverse section of the modern specimen, with flaring sclerenchyma, scale bar is 200μm.

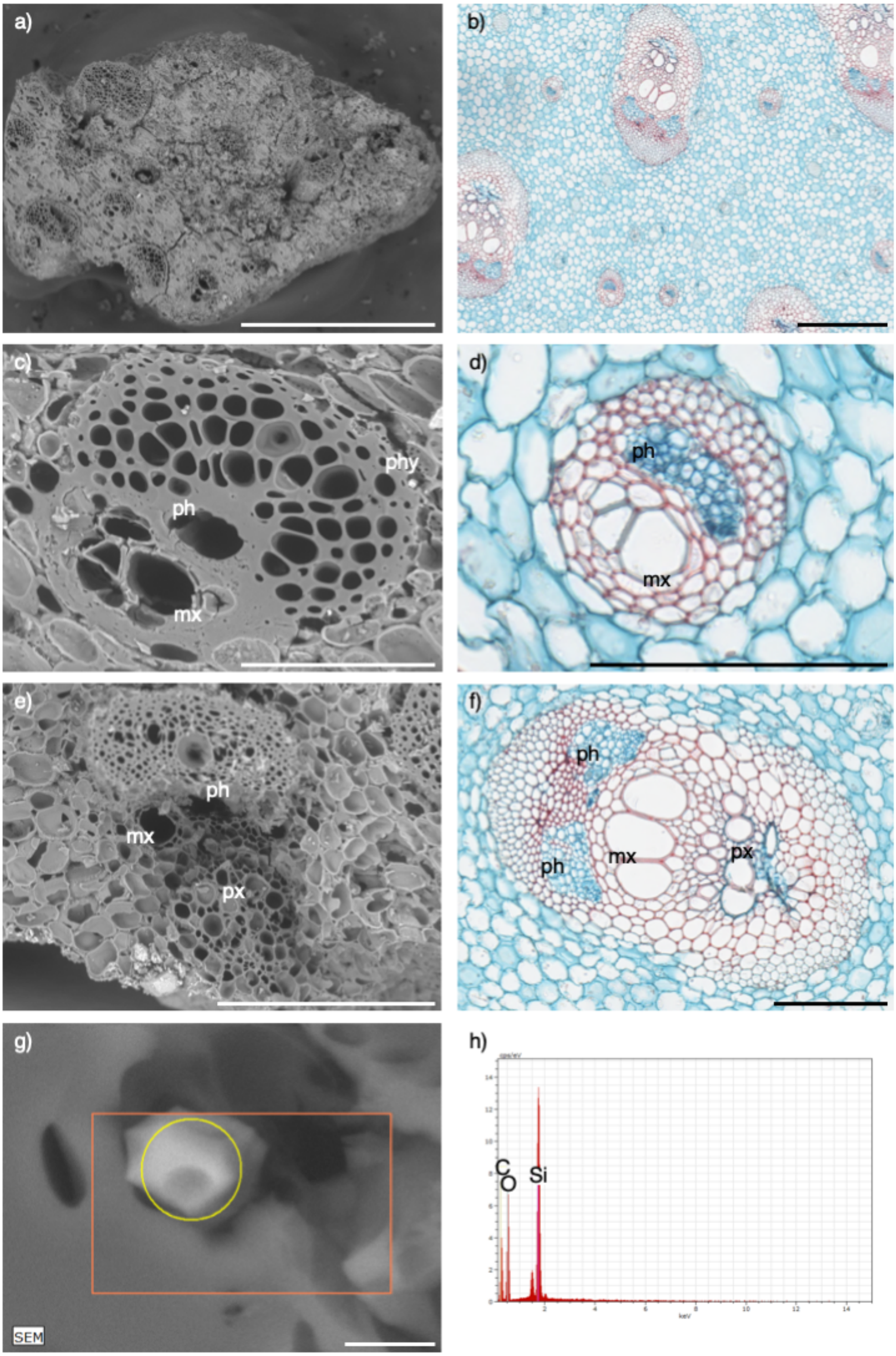

Supplementary Figure 8: Arecaceae family sheath/pith identification proof,

comparing an archaeological palm tissue fragment from C2/44 and C2/38A (left) with a modern reference specimen of *Livistona benthamii* (UQM3223) (right; ph: phloem, mx: metaxylem, phy: phytolith). a) transverse section of the archaeological specimen from C2/44, displaying multiple sizes of fibrovascular bundles, scale bar is 1mm; b) the transverse section of the modern reference specimen, displaying multiple sizes of closed collateral vascular bundles, scale bar is 500μm; c) transverse section of the archaeological specimen from C2/44, displaying a close-up of a small fibrovascular bundle, with multiple metaxylem, scale bar is 100μm d) transverse section of the modern specimen, displaying a close-up of a small fibrovascular bundle, with multiple metaxylem, scale bar is 200μm; e) transverse section of the archaeological specimen from C2/38A, displaying a close-up of a large fibrovascular bundle, with multiple metaxylem and protoxylem, scale bar is 300μm; f) transverse section of the modern specimen, displaying a close-up of a large fibrovascular bundle, with multiple metaxylem and protoxylem, scale bar is 200μm; g) image of a globular echinate phytolith from the archaeological specimen from C2/44, scale bar is 3μm; h) an elemental scan of a globular echinate phytolith from the archaeological specimen from C2/44, clearly identifying it as a silica-based structure (wt.%: 47% O, 42% C, 19% Si).

Supplementary References

1 Northern Teritory Government *Flora NT: Northern Territory flora online*, <http://eflora.nt.gov.au/home> (2013).

2 Nix, H. A. & Kalma, J. D. in *Bridge and Barrier: The Natural and Cultural History of Torres Strait* (ed D. Walker) 61-91 (1971).

3 Torgersen, T. *et al.* Late Quaternary environments of the Carpentaria Basin, Australia. *Palaeogeog., Palaeoclim., Palaeoecol.* **67**, 245-261 (1988).

4 van der Kaars, S., de Deckker, P. & Gingele, F. X. A 100 000-year record of annual and seasonal rainfall and temperture for northwestern Australia based on a pollen record obtained offshore. *J. of Quat. Sci.* **21**, 879-889 (2006).

5 Wightman, G. M. & Andrews, M. R. *Plants of the Northern Territory Monsoon Vine Forests*. Vol. 1 (Conservation Commision of the Northern Territory, 1989).

6 Thomas, R. & de Franceschi, D. Palm stem anatomy and computer-aided identification: The Coryphoideae (Arecaceae). *Am. J. Bot.* **100**, 289-313, doi:10.3732/ajb (2013).

7 Marwick, B. & Florin, S. A. The First Australian Plant Foods at Madjedbebe, 65,000–53,000 years ago. doi:10.17605/OSF.IO/YDUZP, (2019).

8 Clarkson, C. *et al.* Human occupation of northern Australia by 65,000 years ago. *Nature* **547**, 306-310, doi:10.1038/nature22968 (2017).
