# Supplementary material for: The first Australian plant foods at Madjedbebe, 65,000–53,000 years ago
Source: Nat Commun. 2020 Feb 17;11:924. doi: 10.1038/s41467-020-14723-0 (PMC7026095; doi:10.1038/s41467-020-14723-0)
Supplement: Supplementary file 3 — Reporting Summary [file 41467_2020_14723_MOESM3_ESM.pdf]

nature research

Corresponding author(s): S. Anna Florin; Chris Clarkson

Last updated by author(s): Jan 14, 2020

Reporting Summary

Nature Research wishes to improve the reproducibility of the work that we publish. This form provides structure for consistency and transparency in reporting. For further information on Nature Research policies, see Authors & Referees and the Editorial Policy Checklist .

Statistics

For all statistical analyses, confirm that the following items are present in the figure legend, table legend, main text, or Methods section.

n/a	Confirmed	
☐	☒	The exact sample size (*n*) for each experimental group/condition, given as a discrete number and unit of measurement
☐	☒	A statement on whether measurements were taken from distinct samples or whether the same sample was measured repeatedly
☐	☒	The statistical test(s) used AND whether they are one- or two-sided<br>*Only common tests should be described solely by name; describe more complex techniques in the Methods section.*
☐	☒	A description of all covariates tested
☐	☒	A description of any assumptions or corrections, such as tests of normality and adjustment for multiple comparisons
☐	☒	A full description of the statistical parameters including central tendency (e.g. means) or other basic estimates (e.g. regression coefficient) AND variation (e.g. standard deviation) or associated estimates of uncertainty (e.g. confidence intervals)
☐	☒	For null hypothesis testing, the test statistic (e.g. *F*, *t*, *r*) with confidence intervals, effect sizes, degrees of freedom and *P* value noted<br>*Give P values as exact values whenever suitable.*
☒	☐	For Bayesian analysis, information on the choice of priors and Markov chain Monte Carlo settings
☒	☐	For hierarchical and complex designs, identification of the appropriate level for tests and full reporting of outcomes
☒	☐	Estimates of effect sizes (e.g. Cohen's *d*, Pearson's *r*), indicating how they were calculated

Our web collection on statistics for biologists contains articles on many of the points above.

Software and code

Policy information about availability of computer code

Data collection	Excavation: SurveyPro (Total Station); Modern Reference Collection and Ethnobotanical Research: FileMaker Pro
Data analysis	Excavation: SPSS, R, Excel; Archaeobotanical analysis: Excel, NIS Elements, Bruker EDS, Quantax, R, Adobe Illustrator, Tilia

For manuscripts utilizing custom algorithms or software that are central to the research but not yet described in published literature, software must be made available to editors/reviewers. We strongly encourage code deposition in a community repository (e.g. GitHub). See the Nature Research guidelines for submitting code & software for further information.

Data

Policy information about availability of data

All manuscripts must include a data availability statement. This statement should provide the following information, where applicable:
- Accession codes, unique identifiers, or web links for publicly available datasets
- A list of figures that have associated raw data
- A description of any restrictions on data availability

All data is given in table form in the Supplementary Information section of the paper and at a cited data repository: http://doi.org/10.17605/OSF.IO/YDUZP.

Field-specific reporting

Please select the one below that is the best fit for your research. If you are not sure, read the appropriate sections before making your selection.

☐ Life sciences ☐ Behavioural & social sciences ☒ Ecological, evolutionary & environmental sciences

For a reference copy of the document with all sections, see nature.com/documents/nr-reporting-summary-flat.pdf

Ecological, evolutionary & environmental sciences study design

All studies must disclose on these points even when the disclosure is negative.

Study description	Arcaheobotanical analysis of food plant remains from Phase 2 occupation layer at Madjebebe Rockshelter. The remains were quantified using ubiquity. No further statistical analysis was completed on them.
Research sample	All charred plant macrofossils, >1mm in size, recovered from Phase 2 occupation layer at Madjebebe Rockshelter. This includes one hearth (C1-43A) and all Phase 2 spits from Square C2 (C2/46-C2/37). Archaeobotanical recovery occurred via flotation and both the flot and heavy residue portion of the sample, >1mm in size, was analysed.
Sampling strategy	Archaeobotanical recovery occurred via bulk flotation. 100% of the sediment from sampled contexts was floated and analysed. A one-by-one metre square against the back wall of the site was chosen for flotation, as this sheltered square was likely to have the best preservation. All hearths and other features identified during excavation were also floated.
Data collection	Samples were initally sorted using low-powered light microscopy. SAF determined whether charred plant macrofossils were wood charcoal or not. Wood charcoal was not identified as part of this study. All other charred plant macrofossils were characterised and identified by SAF, with additional help from ASF, via high-powered light microscopy and scanning electron microscopy. Sample composition was recorded in Excel.
Timing and spatial scale	Excavation of Madjedbebe occurred in 2012 and 2015. Initial archaeobotanical analysis began in 2013, as par t of an honour's project by SAF. Analysis was continued in 2016 until 2018 as part of SAF's PhD research. Modern reference collection and ethnobotanical research with Mirarr and Nowardekken elders was completed in September 2015, October 2016, June 2017, September 2017, February 2018, July 2018 and December 2018.
Data exclusions	No data was excluded.
Reproducibility	All charcoal and reference collection is available for analysis by other archaeobotanical experts.
Randomization	Samples were allocated into groups via the excavation of 2cm spits.
Blinding	The study was not blinded. Both the archaeobotanical and residue and usewear analysis was, however, completed and worked on by multiple experts in the field.

Did the study involve field work? ☒ Yes ☐ No

Field work, collection and transport

Field conditions	Field work (both archaeological and ethnobotanical/reference collection) was conducted on Mirarr Country in western Arnhem Land. This region has monsoonal wet seasons and subsequent flooding, which can prohibit access to certain localities. Archaeological excavations were carried out in the dry to mediate these conditions. Modern reference collection and ethnobotanical fieldwork, however, required collection over different seasons in different habitats. At some points during the research, helicopters were used to access environments otherwise which were otherwise unaccessable. Howeve, seasonal weather conditions did at times limit the collection of some plant specimens. Plants on the edges of water sources were also collected but with great caution due to salt water crocodile presence in the region. For this reason, some plants were not accessible and attempts have been made to obtain reference material from native nurseries.
Location	The archaeological excavation occurred at Madjedbebe rockshelter, on Mirrar Country, in the Energy Resources Australia Mining Lease. The modern reference collection and ethnobotanical research was conducted in Kakadu National Park, the Arnhem Land Aboriginal Land Trust and the Energy Resources Australia Mining Lease. The latter research occurred across several environments, including sandstone escarpment, monsoon vine forest, woodland, floodplain, freshwater billabongs and estuarine environemnts.
Access and import/export	The Gundjeihmi Aboriginal Corporation was a partner in this research. All necessary permits were acquired, including Permits for access to biological resources from Commonwealth areas (AU-COM2018-391, AU-COM2017-391, AU-COM2016-287); Permits to carry out scientific research in a Commonwealth Reserve (RK909, RK897, RK870); Permit to enter and remain on Aboriginal land or sea adjoining Aboriginal land (research) (DOC160119). All interstate movement of plant specimens was done following State and Commonwealth legislation.
Disturbance	Disturbance was minimised wherever possible. However, collection of modern reference material did require the removal of plant parts. Where possible, this was done in a manner that did not kill the plant.

Reporting for specific materials, systems and methods

We require information from authors about some types of materials, experimental systems and methods used in many studies. Here, indicate whether each material, system or method listed is relevant to your study. If you are not sure if a list item applies to your research, read the appropriate section before selecting a response.

Materials & experimental systems

n/a	Involved in the study
☒	☐ Antibodies
☒	☐ Eukaryotic cell lines
☐	☒ Palaeontology
☒	☐ Animals and other organisms
☐	☒ Human research participants
☒	☐ Clinical data

Methods

n/a	Involved in the study
☒	☐ ChIP-seq
☒	☐ Flow cytometry
☒	☐ MRI-based neuroimaging

Palaeontology

Specimen provenance	Madjedbebe rock shelter, western Arnhem Land, northern Australia. Permission was obtained via an MOU made with the Gundjeihmi Aboriginal Corporation.
Specimen deposition	The specimens in this study are held at the University of Queensland. All future research is at the discretion of the Gundjeihmi Aboriginal Corporation.
Dating methods	Optically stimulated luminescence and AMS radiocarbon dating. Both of these methods are reported in detail in a previous paper: Clarkson et al. 2017 Human occupation of northern Australia by 65,000 years ago. Nature 547(7663):306-310.

☒ Tick this box to confirm that the raw and calibrated dates are available in the paper or in Supplementary Information.

Human research participants

Policy information about studies involving human research participants

Population characteristics	Participants in the ethnobotanical research were both male and female Mirarr and Nowardekken elders. Participants are authors on this paper.
Recruitment	Relevant knowledge holders were identified through the Gundjeihmi Aboriginal Corporation.
Ethics oversight	The University of Queensland, Institutional Human Research Ethics Approval - 2015001177

Note that full information on the approval of the study protocol must also be provided in the manuscript.
